# Identity Work as Ethical Self-Formation: The Case of Two Chinese English-as-Foreign-Language Teachers in the Context of Curriculum Reform

**DOI:** 10.3389/fpsyg.2021.774759

**Published:** 2022-01-10

**Authors:** Anne Li Jiang

**Affiliations:** School of Foreign Languages, Northeast Normal University, Changchun, China

**Keywords:** identity work, ethical self-formation, ethical agency, Chinese EFL teachers, curriculum reform

## Abstract

Curriculum reform urges teachers to constantly reflect on existing identities and develop probably whole new identities. Yet, in the wake of the poststructuralist view of identity as a complex matter of the social and the individual, of discourse and practice, and of agency and structure, teacher identity is a process of arguing for themselves and hence ethical and political in nature. Drawing on Foucault’s notion of ethical self-formation and its adoption by Clarke (2009a) “Diagram for Doing Identity Work” in teacher education research, this 2-year-long case study explores how two Chinese English-as-foreign-language (EFL) teachers engaged in identity work in a changing curricular landscape. The analysis of narrative frames and semistructured interviews reveals the relations between the relative stable and the evolving elements of teachers’ identity work, and the essential role of teachers’ ethical agency based on reflective and critical responsiveness to the contextual reality and the dynamic power relations during the reform. The findings argue for the importance of nourishing teachers’ reflective identity work and ethical agency during the turbulence of educational change.

## Introduction

Curriculum reform is a constant reality in teachers’ professional lives, and English-as-a-foreign-language (EFL) teachers are invariably urged to develop professionally so as to adapt to the challenge of innovation and new curricular (Jiang and Zhang, 2021). However, professional development involves not only continuous learning but also developing new roles, and cultivating new identities. From a post-structuralist perspective, identity is seen as multiple, dynamic, and a site of contradiction and struggle (Norton, 2013). Transformation in teacher identity is, by no means, an easy and linear process. Instead, it is slow, intricate, recursive, and, often, replete with hardship (Vähäsantanen, 2015), largely due to the intimate relationship of identity formation to the discourses and the communities within which teachers work. When seeking to give an account of his/her own identity, a teacher will find this is inevitably influenced by the socially determined norms and discourses, and thus leaving limited space for agency. Therefore, to strive for identity formation or reformation, a teacher has to leverage his/her limited agency to engage in “identity work” (Clarke, 2009a), which, according to Foucault’s notion of ethical self-formation, involves work on the self by the self for self-improvement (Foucault, 1983). This is especially the case for language teachers in the reform context, which often generates new norms, discourses, and power relations. In this sense, how teachers do identity work to actualize identity transformation and professional growth in the stream of curriculum reform deserves in-depth exploration.

In recent years, the tertiary education in China has been undergoing a nation-wide curriculum reform in the wake of the country’s “Double First-Class” initiative. This initiative aims at building a number of world elite universities and disciplines by the end of 2050 in an effort to make China an international higher education power (Ministry of Education, 2017). New missions are thus given to English education. The conventional English-for-general-purposes (EGP)-only curriculum, which targets students’ general English competence, is giving way to a curriculum centered on English for specific purposes (ESP), or its subcategory, English for academic purposes (EAP), which targets more specifically students’ academic and professional English competence for academic study and future career development. This shift has placed a large number of teachers with general English background in a new arena that extends beyond their previous expertise. For one thing, teachers have to grapple with issues such as inadequate disciplinary subject knowledge, catering to students with heterogeneous language proficiency and ESP learning needs, teaching critical thinking, and so forth (Campion, 2016; Jiang et al., 2020). For another, compared with established mainstream areas like general English, literature, and linguistics, the disciplinary status of ESP is underrepresented or even marginalized compared with EGP or other disciplines (Tao and Gao, 2018; Gao and Cui, 2021). ESP teachers thereby tend to be disadvantaged in the implicit institutional power relations.

Considering what is mentioned above, transitioning from EGP to ESP has severely challenged teachers’ previous expertise, beliefs, practices, and, especially, professional identities (Jiang and Zhang, 2021). They have to navigate the tensions between what they are familiar with and the new disciplinary norms and discourses, as well as the shifting power relations. In this process, they need to continuously reflect on and identify who and what they are, as distinct from who and what they are not, and to make ethical choices in the contingency of power relations. This means, in order to realize identity transformation and professional growth, these teachers need to exercise an ethical agency to engage in identity work as ethical self-formation. Aiming at unpacking this process, this case study draws on Foucault (1983) notion of ethical self-formation, especially its representation as Clarke (2009a) ethico-political diagram of identity work as the conceptual framework to guide the exploration of how two Chinese university English teachers ethically and agentively engage in identity work in the transition from EGP to ESP. Specifically, it attempts to answer a central question: How do the two university teachers transform their identities, shifting from EGP to ESP?

## Literature Review

### Understanding Language Teacher Identity Formation as Ethical Self-Formation

Teacher identity can be broadly understood as teachers’ sense of themselves and the image that they present to others (Day, 2011). From a poststructuralist perspective, identity formation has an intimate relation to the discourses of the communities where teachers work, and it is partly determined by the preexisting discourses. However, teachers as agentive beings are still able to make ethical choices to resist what is unfavorable, and thrive on new linguistic and social resources (Clarke, 2009a). On this point, Foucault’s notion of ethical self-formation offers an important perspective. He viewed ethics as “a process in which an individual delimits that part of himself or herself that will form the object of his or her moral practice, defines his or her position relative to the precept he or she will follow, and decides on a certain mode of being that will serve as his or her moral goal” (Foucault, 1992, p. 28). Thus, ethical self-formation is regarded as a sort of work, an activity requiring reflective and agentive work on the self by the self. Built on this notion, and recognizing that identity is formed at the nexus of the individual and the social, Clarke (2009a) further elaborated the connection between teacher identity and ethics, agency, and reflection. He postulated that facing social discourses, cultural conventions, and pervasive power relations, teachers can make ethical judgment, engage in reflection, and exercise ethical agency to shape and reshape their identity, i.e., to conduct identity work as a process of ethical self-formation.

In the literature of language teacher identity, the cases where teachers engage in identity work as ethical self-formation through agentively and reflectively working on themselves can be found in a number of studies. One line of research pertains to how language teachers’ identities are forged in relation to their gender, race, and the wider power, ideological and sociopolitical structures. For instance, Barkhuizen’s longitudinal investigation of a female Tongan EFL teacher’s identity trajectory revealed how the teacher made ethical choices and worked on herself through investing in imagined identities, which finally empowered her to “agentively position herself in a social-local world that provides her with the capital that she and her community need” (2016, p. 678), instead of becoming complicit with the normative TESOL ideology. Zhang and Zhang (2015)’s ethnographic study showcased how two non-native English-speaking teachers (NNESTs) from China justified the legitimacy of their professional identity through taking stock of their professional expertise in an institutional discourse that favors native English speakers to be standard practitioners of English education. This study aptly elucidated the primacy of agency and the constitutive role of reflection in language teacher’s identity work against unfavorable institutional discourses and imbalanced power relations between native and non-native English teachers. In a similar vein, Song (2016) research explored how a group of NNESTs, confronting the questioning of their lack of native-like expertise, managed to actualize identity transformation against the dominant discourse through agentively reflecting on their emotions and seeking possible alternatives by confronting and transforming them.

Another line of research concerning language teacher identity construction through social participation also demonstrates how teachers forge their identities as ethical self-formation in competing discourses and power dynamics. In Lee (2013) study, for instance, facing the tensions between their new pedagogical approach acquired from the teacher education program and the established institutional discourses of writing teaching rules, beliefs, and practices, teachers positioned themselves as agents of change, and kept implementing new teaching methods and making improvement through constantly reflecting on their teaching practices. In this way, they acquired new discourses to make sense of themselves and new ways to enact their identities as writing teachers. In the Chinese university context, Xu (2013) study documented how teachers transformed their identities from “imagined” to “practiced” during the transition from pre-service to novice teachers. His study found that agency contributes considerably to the development of identity. Likewise, despite the contextual constraints, including the rigid school curriculum and the imbalanced power relations between teachers and the university researchers, the teachers in Yuan and Burns (2017) research took agentive boundary-crossing actions, which enabled them to negotiate meanings for their expanded identities.

On the whole, previous language teacher studies are replete with cases, which indicate teachers’ ethical identity work. However, research specifically drawing on the conceptualization of teacher identity work as ethical self-formation is still scarce, with a limited number of studies focusing on, for example, pre-service teachers (Clarke, 2009b) and teacher educators (Miller et al., 2017). Given identity’s contingency on social discourses and power relations, more research on language teacher identity work should be conducted to unpack how teachers construct and reconstruct their identity through agentively engaging in identity work as ethical self-formation.

### A Necessity to Explore Teachers’ Identity Work as Ethical Self-Formation in the Reform Context

In the reform context, teachers often draw on their identities to exercise an agency during educational change, which, in turn, maintains or transforms their identities (Eteläpelto et al., 2013; Vähäsantanen, 2015). However, educational reform may often put teachers’ identities and agency at stake due to heightened expectations, broader demands, increased accountability, and complex power dynamics. Teachers may feel vulnerable and constraint agency due to inconsistency between their higher moral goals of teaching, core aspects of their professional identities, and the negative political tone and mandates of the reform (Lasky, 2005). They may also lose agency and feel emotionally impaired when failing to derive meanings because their own identity beliefs and practices are incommensurate with what the reform promoted (Liu and Xu, 2011).

In some circumstances, it seems that teachers are committed to certain identities, which are so crucial as to energize them to embrace the new reform enterprise and to engage in career development even when being marginalized in the institutional power relations and shifting structure, as shown in Tao and Gao (2018) research. The so-called identity commitment is also found to be boosters for teachers to actively exercise agency to face the challenges of reform and to open up possibilities for new identities and future professional development. A case in point is Gao and Cui (2021) research. In their research, the focal teacher has navigated evolving identities, such as giver, explorer, learner, and so forth over time, signifying the discontinuity of identity (Akkerman and Meijer, 2011). Yet, the commitment to being an “authority in the classroom” providing knowledge for students plays an essential role in contributing to the continuity of her identity during the curriculum reform. This being said, the core identity elements may also be malleable and mediated by the reform context. Jiang and Zhang (2021) study, for instance, elucidated how the new curriculum changed some teachers’ ontological and epistemological beliefs about teaching and learning, and consequently managed to transform and expand their professional identities.

By and large, teachers’ perceiving, adapting, and implementing the reform is invariably accompanied and influenced by committing to or challenging and reconstructing their existing identities. It involves an ongoing interaction between an individual and the social, and between one’s agency and the new discourses and power dynamics caused by the reform. Even in general situation, considering the social shaping of one’s identity, the scope for exercising agency in identity formation or reformation is not unlimited (Clarke, 2009a). As educational reforms usually generate new discourses and power dynamics, teachers need to continuously reflect on and identify who and what they are, as distinct from who and what they are not, and to make ethical choices in the contingency of power relations. For this reason, it is necessary to explore how teachers engage in identity work as an ethical self-formation to shape and reshape their identities during the reform. Such exploration will help to shed light on how language teachers agentively and reflectively negotiate meanings and realize professional development in times of curricular change.

## Theoretical Framework

**FIGURE 1:**
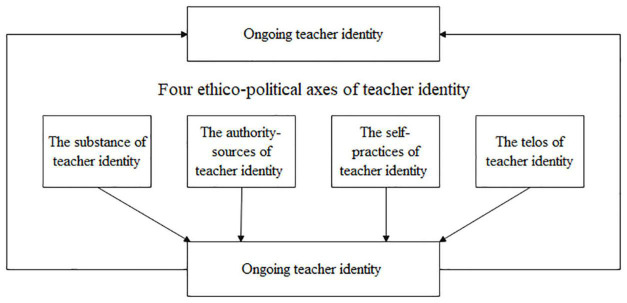
A diagram for doing identity work.

To explore how Chinese university English teachers ethically and agentively engage in identity work during the curricular reform, the current study draws on Foucault (1983) notion of ethical self-formation, especially its representation as Clarke (2009a) ethico-political diagram of identity work as the conceptual framework. This diagram consists of four axes (see Figure 1).

The first axis is the substance of teacher identity, which refers to the aspects of the teachers’ selves that they utilize to constitute their teaching selves. It may include all those factors that constitute a teacher’s identity, such as one’s emotions, characters, beliefs, knowledge, and skills, among others. The second axis is the authority sources of teacher identity (also referred to as a mode of subjection), which concerns the attitudes, beliefs, and codes of behavior and discourses that teachers consider to be sources of authority that inform them as to how to be teachers. The third is the self-practices of teacher identity, which can include a range of physical and mental techniques and practices used to fashion and shape teachers’ selves. The last is the telos of teacher identity, or the goals and purposes that teachers have concerning their teaching selves. To apply this diagram to analyzing a teacher’s identity work for illustrative purpose, Clarke (2009a) offered an exemplar case of a teacher who used his “personal character” as the substance of his teaching self, his belief of life-long learning and continuous improvement as the authority sources to judge his ethical teaching behavior, his classroom-based teaching practices as the focus of his self-practice as a teacher, and the idea of “teaching as a meaningful and rewarding job” as the telos of his/her teacher identity.

## Materials and Methods

### Context and Participants

The present case study is set in a prestigious medical university in northeast China. Aiming at cultivating talents with solid professional knowledge foundation and high English proficiency for academic and professional purposes, this university replaced its long-established EGP-only curriculum with an ESP-dominant curriculum several years ago. The stipulated curriculum objective is to develop students’ English competence in handling general academic tasks, and get them prepared for subject-matter learning in English (i.e., medical courses are taught in English as the medium of instruction, “EMI courses” for short hereafter) and future research and professional work. Apart from the first semester of EGP study, students are offered three semesters’ ESP courses, which were completely new to the teachers of the English department. Holding postgraduate degrees mostly in English literature and applied linguistics, and having been teaching EGP all along, these teachers had no idea what and how to teach at the beginning of the reform. Since ESP is distinct from EGP in terms of content, methodology, and objective (Basturkmen, 2010), teachers were challenged to create new syllabus, design courses, and prepare appropriate teaching materials that can meet students’ specific ESP learning needs. Meanwhile, for the reason that students usually have more subject-matter knowledge, English teachers somewhat lost their previous status of “knowledge authority” in the ESP classroom. In addition, despite the university’s top-down curriculum reform initiative, ESP as a new product in the focal university was unrepresented than EGP or other disciplines. Overall, English teachers were under great pressure implementing the new curriculum.

After obtaining ethics approval from the author’s university ethics committee, two female ESP teachers, Wang and Zhao (pseudonyms), who the author got to know at a local language teaching conference and has been in touch ever since, were invited to participate in this longitudinal study. They were purposefully selected for three reasons: First, they characterize the English teacher demography of the specific research context, since, in the focal university, approximately 95% of the staff of the English department are female. Second, both teachers had more than 10 years of EGP teaching experience before shifting to ESP and were recognized as expert EGP teachers by the university. Lastly, neither of them had received any training or doing research in the field of ESP; therefore, the challenges inherent in the distinctive linguistic and pedagogical features of ESP were paramount (see Table 1 for their demographic information). Like their colleagues in the same department and most other EGP teachers who are new to this field of ESP, Wang and Zhao have been struggling for identity transformation and professional development. How they do identity work as ethical self-formation in the new landscape deserves in-depth exploration.

**TABLE 1:** General demographic information of the focal participants.

Name (pseudo)	Gender	Age	Education	Prof title	Years of EGP teaching	Years of ESP teaching by the end of this study
Wang	Female	46	M.A. in English literature	Professor	20	3
Zhao	Female	39	M.A. in applied linguistics	Associate professor	13	3

### Data Collection and Analysis

This study adopted a narrative inquiry approach. Narrative inquiry, also referred to as the study of experience as a story, holds the view that humans lived storied lives, and the story is a portal through which a person enters the world and make meanings (Connelly and Clandinin, 2006). Since “the nature and meaning of experiences are difficult to observe directly and best understood from the perspective of those who experience them” (Barkhuizen et al., 2014, p. 8), a narrative approach is especially valuable. Life stories and narratives are not simply about accounts of experiences but also about how people ascribe meanings to those experiences (Clandinin et al., 2007). As such, the study of teachers’ stories and narratives can help researchers to understand the complexity of their identity construction (e.g., Tsui, 2007). For the present study, the necessity and benefit of adopting a narrative inquiry approach are 2-fold. On the one hand, the idea of teachers’ identity work as ethical self-formation foregrounds the post-structuralist approach to identity as discursively constructed (see Clarke, 2009a); it is, therefore, necessary to focus on participants’ own stories and narratives of their experiences. On the other hand, since the research aim is to understand how the focal teachers engage in identity work to transform their identities shifting from EGP to ESP, the narrative approach can help to illuminate their lived experiences of the transition by connecting their past to their present as well as future lives and identities.

Specifically, the data came primarily from two sources: narrative frame journals and semistructured interviews. A narrative frame is a written story template consisting of a series of incomplete sentences and blank spaces of varying lengths. The aim is for participants to produce a coherent story by filling in the spaces (Barkhuizen, 2011). The merit of using a narrative frame for this study is that the stories it helps reconstruct can introduce the three-dimensional life space of teachers’ life histories (Xu and Connelly, 2009), and offers specific contextual and relational background for this case study. The template was framed according to the four axes of a diagram of identity work (see “Appendix” for the details). In this way, the stories reconstructed could reflect teachers’ action, agency, and reflection in relation to their identity work embedded in specific events. Each teacher was asked to write two journals through completing the sentence starters and adding anything they thought necessary. The one written at the beginning of the research was expected to reflect their reflection and stories related to EGP teaching, which helped to generate subsequent interview questions. The other one written at the end was expected to reflect their belief and experience of ESP teaching, possibly with some thoughts inspired by the interviews during the research. The journals were written in the teachers’ L1 so as to guarantee accuracy and better expression. Wang’s two journals average 1,032 words for each, while Zhao’s average 926 words for each.

Apart from the journals, four rounds of semistructured interviews, each lasting about 1 h, were conducted individually with each participant. The first and last interviews took place right after the narrative frame journal was finished, and the other two interviews were done in between. The topics of the interviews concerned the participants’ life history about past and present learning and working experiences, their reflections and practices on their identity work, changing from an EGP to an ESP teacher, and imagination of future development (see “Appendix,” for example, questions). Both the narrative frames and interviews were done in Chinese, the participants’ native language, and later transcribed and translated into English by the author.

Thematic analysis was utilized for both the narrative frame journals and the interview data. Teachers’ responses to the narrative frames were extracted and combined to form stories. These stories were then analyzed thematically according to the *a priori* analytical categories (Yin, 2009) defined based on the ethico-political diagram of teacher identity. Analysis of the interview data involves a process of open and axial coding (Huberman and Miles, 2002). The two participants’ interview responses were first read and reread to identify emerging codes. The identified codes were then grouped into four themes according to the four axes of the ethico-political diagram. The codes and themes were compared and juxtaposed both within and between the participants to establish interconnections, revealing their identity work amid the reform (see the “Appendix” for themes, codes, and example quotes). The coding was carried out by the author and an experienced researcher. After independent coding, they jointly compared and discussed their own interpretation until all the disagreements were resolved. The narrative frame journal data were triangulated with the interview data through comparing and juxtaposing the codes and themes of the two sets of data. In this way, the trustworthiness of the analyses was enhanced.

## Findings

Analysis of the narrative frame responses and interview data shows that the two teachers have agentively worked on themselves to transform their identities and practices to adapt to the new curriculum. Such transformation is embodied in the four ethico-political aspects of their identity work, as elaborated in the following section.

### The Substance of English-as-Foreign-Language Teacher Identity

From Wang and Zhao’s narrative frame responses, two forms of substances that they used to constitute their teaching selves can be identified, including “knowledge of the English language and teaching” and “self-concept.” These themes are consistent irrespective of the difference between EGP and ESP teaching (see Table 2 for example responses).

**TABLE 2:** The substance of teacher identity.

Theme	Selected responses to sentence starter 1 in both EGP and ESP frames
Knowledge of English language and teaching	My solid knowledge foundation of English language and my own English proficiency (Wang-EGP) My capability to teach English well. I have the ability to make my teaching appealing, much as I know how to cater to the diverse tastes of my students (Zhao-EGP) My expertise in applying various teaching methods in line with the needs of the lesson” (Wang-ESP)
Self-concept	My faith in myself as a good English teacher and my love toward the students (Wang-EGP) My enthusiasm for this career and my belief that I’m doing something meaningful and I can do well (Zhao-ESP)

As shown in Table 2, for both teachers, language knowledge [e.g., “my solid knowledge foundation of English language and my own English proficiency” (Wang-EGP)] and teaching skills [e.g., “my expertise in applying various teaching methods in line with the needs of the lesson” (Wang-ESP)] and the self-concept of being a good teacher [e.g., “my faith in myself as a good English teacher and my love for the students” (Wang-ESP)] and the belief that teaching is meaningful [e.g., “I’m doing something meaningful” (Zhao-ESP)] have remained at the core of their identity formation and transformation during the shift from EGP to ESP teaching. Recalling EGP teaching experience, Wang and Zhao were quite confident about their role as the authority of knowledge and their pedagogical practices, as evident in the interview excerpts below:

Excerpt 1: *Wang interview 1 early year 1*

*I would say I am a qualified teacher, or a knowledge authority, I mean, whenever students have learning problems or difficulties, they come to me, and they can always get a solution.*

Excerpt 2: *Zhao interview 1 early year 1*

*The classroom was my stage. I was so much empowered by the knowledge I had. I enjoyed every moment when students were fascinated by the knowledge and learning skills I shared with them. I felt like I unleashed a certain potential that I hadn’t been aware of.*

In addition, the aspiration to be a good teacher, or the self-concept of being a good teacher, is also crucial for his or her identity. Students’ progress, not only academic but also as whole persons, seems to be a key criterion for them to make judgment about whether they are good teachers and doing meaningful work, as indicated in Zhao’s accounts:

Excerpt 3: *Zhao interview 2 end of year 1*

*As an English teacher, I embrace a utilitarian objective of English education. My students’ excellent performance, either in exams, contests, EMI classes, or whatever, always gives me a sense of happiness and achievement. Whereas, as a teacher, I hope I can orient students to continue their will willingly, which will make me feel I’m doing something really meaningful.*

However, when they first taught ESP, the abovementioned substance of their identity was affected, which then led to their identity crisis. First, Wang and Zhao were emotionally struggling and felt at a loss due to a lack of ESP-specific language and pedagogical knowledge to effectively handle the new curriculum. What is most agonizing to them is students’ dissatisfaction. During the first semester of medical English teaching, Zhao was frustrated because her frequently used teaching method of “simply translating and paraphrasing” was not well received by her students. And the end-of-semester students’ assessment of teaching revealed their prevalent complaints that the course content was detached from their real needs, especially for EMI learning. Zhao recalled:

Excerpt 4: *Zhao interview 3 middle of year 2*

*I don’t fear failure, but I fear methodological fallacy, narrow-mindedness, and the inertia to cling to past glory. The last thing I would do is to waste my students’ time by forcing them to engage in a learning experience that deviates from their actual needs…*

Wang mentioned similar pedagogical predicament in her ESP teaching, which, for a long time, was dominated by teacher-front lectures with little interaction with students. “It felt like a setback of my professional life,” she reflected, “I began to doubt myself as a teacher.”

What further exacerbates their identity crisis is the negation and lukewarm attitudes of the university leaders and subject-matter teachers in the first 2 years when the new curriculum was implemented. Wang’s recounts below, in a certain sense, indicate how the negative discourse of the others impacted on the substance of her identity, i.e., “to be a good teacher,” and thus led to her identity crisis. What is also implicated is the disadvantaged position of the ESP teachers and their enterprise in the power relations of the institution.

Excerpt 5: *Wang interview 1 early year 1*

*At that time, university leaders were not satisfied with us. Our qualification for teaching ESP was questioned, and the worth of the course was severely under-evaluated. Some subject-matter colleagues, especially those experts who possess a greater say, even expressed it is not necessary to shift to this new curriculum. I was low in moral and even began to doubt myself as a good teacher.*

### The Authority-Sources of English-as-Foreign-Language Teacher Identity

The authority sources of teacher identity primarily concern the norms or moral obligations by which teachers can weigh their identity work (Miller et al., 2017). Wang and Zhao’s authority sources of identity have changed with the shift from EGP to ESP.

As can be seen in the narrative frame responses in Table 3, when teaching EGP, they felt more obliged to effectively transmit knowledge to students [e.g., “I was a teacher who transmitted English language knowledge and cultural tips” (Wang)] and guide their learning [e.g., “I often directed students to learn necessary English skills” (Zhao)]. In contrast, ESP teaching has changed this obligation. They began to judge their ethical work on themselves with a different set of authority sources. To meet students’ needs becomes a new mode of subjection [e.g., “students should be able to use English for study and work after attending the ESP courses” (Wang)]. Their interview responses also support this point, as Zhao commented: “It is not what you think is useful or important that matters—it is students’ real learning needs and the needs of the discipline.” They no longer stick to any fixed teaching objectives or content; instead, they would make frequent adaptation based on regular learning needs assessment.

**TABLE 3:** The authority sources of teacher identity from English-for-general-purposes (EGP) to English-for-specific-purposes (ESP).

Theme	Selected responses
**Theme for EGP teaching**	**Selected responses to sentence starters 2–4 in EGP frame**
Transmit knowledge	I was a teacher who transmitted English language knowledge and cultural tips (Wang) I believed my function was to transmit language knowledge in a way that was easy to understand and remember (Zhao)
Guide learning	I often directed student to learn necessary English skills (Zhao) I believed students needed the teacher to guide them with regard to the learning content and methods (Wang)
**Theme for ESP teaching**	**Selected responses to sentence starters 2–4 in ESP frame**
Meet students’ needs	Students should be able to use English for study and work after attending the ESP courses (Wang)
Engage in boundary-crossing process	ESP needs collaboration between English teachers and subject matter teachers (Zhao)
Serve students	I see myself as a learning facilitator (Zhao) I see myself as facilitating students to know how to learn ESP (Wang) ESP curriculum should serve the needs of content learning in the disciplinary areas (Wang)

Moreover, as shown in teachers’ narrative frame responses in Table 3, teachers regard teaching and learning ESP as a boundary-crossing process, where the boundary between English teachers and subject-matter teachers is crossed [e.g., “ESP needs collaboration between English teachers and subject matter teachers” (Zhao)]. Furthermore, revealed in the interview is that the boundaries between English teachers and students, and between English and disciplinary subjects are also constantly blurred and crossed, as indicated in Zhao’s words:

Excerpt 6: *Zhao interview 3 middle of year 2*

*The university discourse tends to relegate ESP teaching completely to the responsibility of English teachers, and teachers must be knowledge authorities. I used to think so too. However, teaching ESP offers a new perspective. Knowledge can and should be co-constructed by the teacher and the students, and by English teachers and subjective-matter teachers; it should be a teamwork, where sharing is always important.*

Meanwhile, they value the idea of teaching as a way to serve the students. They would like to act as a learning facilitator to facilitate students to master ESP learning methods, and make ESP courses more useful for preparing the students for their subject-matter EMI learning, as shown in the narrative frame responses [e.g., “I see myself as a facilitating student to know how to learn ESP” (Wang)]. All these have formed a new set of criterion for the teachers to judge their identity work.

### The Self-Practices of English-as-Foreign-Language Teacher Identity

This dimension concerns all kinds of techniques and practices teachers use to shape and fashion their teaching selves. These techniques often require a certain degree of intentional change of the self that may hold the potential for broadening one’s sense of identity and for changing the repressive identity that is socially designated (Miller et al., 2017). Shifting from EGP to ESP, Wang and Zhao’s practices changed dramatically as manifested in their narrative frame journals.

When teaching EGP, teachers mainly form their identities based on classroom teaching practices. As the example responses in Table 4 suggest, they focused more on teaching language knowledge and training students’ communicative and practical skills [e.g., “I would teach grammatical and textual knowledge” (Zhao), “I would lead students to do a lot of translation and writing exercise” (Wang)]. Comparatively, teaching ESP created a new space for them to engage in extended or new activities. For lack of subject-matter knowledge, Wang and Zhao, and probably their other ESP colleagues, have often encountered cases where the course content involves too much disciplinary knowledge beyond their capacity to handle. This often leads to classroom emergencies where teachers made mistakes or could not answer students’ questions. Sometimes, students can become “particularly picky or critical because they usually have more subject-matter knowledge and thus more professional with respect to the content of the teaching material” (Wang’s interview quote). Reflecting on such reality and the features of the course, Wang and Zhao have enacted ESP teaching and learning as a collaborative process involving English teachers, subject-matter teachers, and students. As evident in their narrative frame responses in Table 4, teaching is no longer in the sole charge of the English teachers. Students are motivated to help collect teaching materials and share subject-matter knowledge [e.g., “I often invite students to select and compile teaching materials with me” (Wang), “I set up an online learning group with the students, so we can solve subject-matter-related language problems together” (Zhao)], and subject-matter teachers are involved in planning lessons, providing teaching materials and offering professional consultancy (e.g.).

**TABLE 4:** The self-practices as an EGP vs. an ESP teacher.

Theme	Selected responses to sentence starter 5 in EGP frame
Teach language knowledge	I would teach grammatical and textual knowledge (Zhao) I used to teach vocabulary and reading comprehension (Wang)
Train communicative and practical skills	I often trained students’ listening and speaking skills (Zhao)
	I would lead students to do a lot of translation and writing exercise (Wang)
	**Selected responses to sentence starter 5 in ESP frame**
Collaborate with students	I often invite students to select and compile teaching materials with me (Wang) I set up an online learning group with the students, so we can solve subject-matter related language problems together (Zhao)
Collaborate with subject-matter teachers	I will consult subject-matter teachers about subject knowledge, asking them to verify my understanding or direct me to proper learning materials (Zhao) I ask EMI teachers to provide teaching materials (Wang)
Learn for development	I’m determined to learn related subject-matter knowledge for better teaching practice (Zhao) I’m a learner myself, and I must always learn new things to realize self-growth (Wang)

Concomitantly, since ESP teaching required them to come to terms with new experiences and new understandings of their professional roles and functions, the two focal teachers have also initiated and engaged in expansive learning for self and collective development. Reflecting on her not-so-successful ESP teaching experience in the first semester, Zhao was determined to learn with and even from her students, as she recounted:

Excerpt 7: *Zhao interview 2 end of year 1*

*I told my students that I will learn with them and, sometimes, even from them, because ESP is a challenge for me too, especially in terms of the medical science knowledge it involves. For instance, once, I asked my students to give me English lectures on the structure of the human heart and the mechanism of diabetes.*

Wang, as the leader of the ESP teaching panel, not only actively learned relevant subject-matter knowledge herself but also encouraged her fellow teachers to attend subject-matter courses and network with EMI teachers. A good reason is that this is a most feasible means to address ESP teachers’ needs for learning subject-matter knowledge and designing ESP courses when there was a lack of institutional resources for teacher training and explicit course guidelines. Wang described the predicament as follows:

Excerpt 8: *Wang interview 3 middle of year 2*

*The university simply stipulated the implementation of ESP teaching in a top-down manner without providing any definite course guidelines. They wanted us, English teachers, to present appropriate syllabus, curriculum design, methods, and materials. However, unlike EGP, ESP was new to us, as it was to most of our colleagues in other universities. We needed training; we needed to do needs analysis, but funding or policy support was hardly available. Compared with other subject-matter faculties, we are low in the university power hierarchy, somewhat marginalized” and the resources at our disposal are limited. So, to engage in in-house training through learning from and networking with subject-matter colleagues was the best solution at the moment.*

The words “low and marginalized in the university power hierarchy” in the above quotes partially revealed the ESP teachers’ status in the focal university’s discourse system. When the university leaders proposed that a synergy should be built between ESP courses and EMI courses so that the former can better facilitate the latter, some ESP teachers were reluctant and resistant. “They felt like being pushed to a subordinate status, like sort of teaching assistants working for our subject matter counterparts,” Zhao said in the interview. However, believing that an adjunct model of ESP is, in effect, beneficial to the students, Zhao took the lead to practice this model in her own class and received positive comments from students and the university leaders. Seeing this, her fellow teachers were more motivated and confident about the new practice, as Zhao narrated:

Excerpt 9: *Wang interview 4 end of year 2*

*My little success was a stimulus to our teaching panel and other ESP teachers. Those who were suspicious of an adjunct ESP model had more confidence in its feasibility and benefits for students. Since then, most of us have been more enthusiastic about learning subject-matter knowledge and collaborating with students and subject-matter colleagues. We have formed an ethos of learning so as to become better ESP teachers.*

### The Telos of English-as-Foreign-Language Teacher Identity

The telos concerns the ultimate goal or purpose of teachers’ teaching selves. It is the accomplishment of, or a quest to achieve an accomplishment of the sort of a person one wishes to be (Niesche and Haase, 2010). The narrative frame responses demonstrate the consistent and evolving elements of the focal teachers’ telos from being an EGP to an ESP teacher. What is consistent is that they always aim to be expert teachers. What has changed is the nature of the motivation underneath this aim.

When teaching EGP, they aspired to gain external recognition as expert teachers, as shown in Wang’s narrative frame response in Table 5, especially the praise and positive evaluation from the university authorities and peers, as Zhao described in the interview:

**TABLE 5:** The telos of teacher identity from EGP to ESP.

Theme	Selected responses to sentence starter 7 in the ESP frame
An expert EGP teacher → an expert ESP teacher	I aimed to become an expert in teaching college English; now my goal is to provide qualified ESP teaching to my students. I hope in the future, with years of experience I can make an expert ESP teacher (Wang)
To gain external recognition → To realize and maximize self-growth	I aimed to consistently obtain positive evaluation and praise from university authorities and the students. Now my goal is to improve my capacity as an ESP teacher through continuous learning. I hope in the future I can maximize my self-worth as an expert ESP teacher (Zhao)

Excerpt 10: *Zhao interview 1 early year 1*

*It used to be a great honor for me to be awarded the title of “Excellent Teacher of the Year” or praised by the teaching quality monitors appointed by the university to regularly check on our teaching. To be an expert teacher, you must gain the recognition of the university authorities.*

When it comes to her ESP teaching experience, Zhao still strives to become an expert teacher. However, she is no longer motivated by the desire to get recognized by the university or peers. Instead, she is driven by the desire to realize self-growth, as she expressed in her narrative frame response: Now my goal is to improve my capacity as an ESP teacher through continuous learning; I hope in the future I can maximize my self-worth as an expert ESP teacher.” Wang revealed similar telos in the interview:

Excerpt 11: *Wang interview 4 end of year 2*

In the past, I aimed to meet various criteria stipulated by the university. Now, my goal is to realize self-development while catering to students’ learning needs. In the future, I hope I can contribute more to the development of ESP as a discipline and enrich my self-worth as an ESP teacher.

In sum, the above findings show that, apart from the substance of teacher identity, all the other three dimensions of the identity of Wang and Zhao have witnessed changes. Facing the new curriculum and its resulting challenges, they have changed the substances of their identity, their teaching practices, and even their ultimate goals as English teachers. And these changes occurred mostly out of their own initiative and based on their own reflection on practice. In this sense, these changes also embody how Wang and Zhao have agentively engaged in identity work to transform their identities.

## Discussion

Built on Foucault’s notion of ethical self-formation and Clarke (2009a) ethico-politics of identity work, the way two Chinese university English teachers transformed their identities and adjusted their professional practices in the context of curriculum reform is explored to see how they have worked on their teaching selves, shifting from EGP to ESP teaching.

In the present study, the process of Wang and Zhao’s reshaping their identities in the reform conditions has revealed what is relatively stable and what is changing and malleable among the four components of their identity work. The substance they used to constitute their teaching self-knowledge of the English language and teaching and self-concept-remains constant irrespective of what courses they are teaching. In contrast, the other three aspects have all evolved with the progress of the reform. There seems to be some causal relationship between the unchanged and the changed. For one thing, it is just because the focal teachers regard their language and teaching knowledge and their self-concept (i.e., being a good teacher, loving students, and teaching as a meaningful career) as the fundamental constituents of their teaching identity that they adjust the way they make judgment of their action (i.e., whether their teaching can meet students’ needs) and their practices (i.e., collaborate with students and subject-matter teachers). For another, in a certain sense, the substance of being a good teacher is in common with his or her telos of “being an expert EGP or ESP teacher.” To maintain the substance and achieve the telos drive the teachers to transform or extend their identities: from being an expert EGP teacher to an expert ESP teacher, a learner and a cross-boundary collaborator. Based on this causal relationship, the unchanged component of their identity work can be regarded as the impetus that promotes the interaction between established and emerging identities (Arvaja, 2016) and keep the continuity of identity in changing contextual conditions as reported in Gao and Cui (2021) research.

Moreover, the unchanged substance and the evolving telos of Wang and Zhao’s identity work also function similarly to the so-called identity commitment, which is crucial to gauging teachers’ agentive choices and actions facing contextual opportunities or constraints (Tao and Gao, 2018). Aside from the challenges of ESP *per se*, the ESP teachers in the focal university were doing identity work within implicit power relations and social positioning (Lai et al., 2016). Despite their disadvantageous power status in the institutional hierarchy and marginalized disciplinary position, Wang and Zhao still took agentive actions to “exercise some sort of power” (Lasky, 2005, p. 913). They replace their old authority sources with new ones and adapt their teaching practices according to the nature of ESP courses and students’ real needs, which evidences the idea that “there is always room for maneuver” (Priestley et al., 2012, p. 210). Their self-practice of ESP teaching as collaboration with students and subject-matter colleagues aligns with their ethical judgment or belief that ESP teaching should be a boundary-crossing enterprise. Such practices subsequently gave rise to new identity possibilities. All this makes the point that their agency of conducting identity work in the reform climate was inextricably interlaced with and motivated by their reflection of the authority sources, or beliefs about the right ways to be a teacher (Lasky, 2005).

A crucial point of seeing teachers’ identity work as ethical self-formation is to understand how teachers construct their identities while critically constraining or enabling other possibilities and to consider possible alternative aspects, comprising different identities (Clarke, 2009a). The reform endows Wang and Zhao with an opportunity to reflect on what has made them who they are and set up alternative possibilities. Shifting to ESP, they became aware that their previous identities as expert EGP teachers were highly contingent on the recognition and praise of the university (see “Excerpts 10, 11”), i.e., the institutional normative discourse. Through implementing ESP, however, they agentively strived “to develop and transform oneself and to attain a certain mode of being” (Foucault, 1997, p. 282). Rather than clinging to the culturally rooted image of teachers as the authority of knowledge (Song, 2016), they come to embrace the new idea of “teaching as expansive learning.” And their telos to become an expert ESP teacher is no longer subject to the ideal role and behavior designated by the institutional discourse (see “Excerpt 10”), but rather to realize and maximize self-growth. All these are a legible proof of their “care of the self” [Foucault (1982), as cited in Clarke (2009a)].

Apart from “care of the self,” Wang and Zhao’s identity work also demonstrates their critical responsiveness to the new curriculum discourses and power dynamics. Zhao’s stepping away from the institution’s discursively constructed stereotype that teachers are knowledge authority (as in “Excerpt 6”) and Wang’s taking the lead to implement an adjunct-ESP model despite her colleagues’ reluctance are good cases in point. Actually, such critical responsiveness can find traces in many cases, such as in Zhang and Zhang (2015)’s study where the two NNESTs who practiced against an institutional discourse, and in Barkhuizen (2016) study where the focal teacher did not conform to the normative TESOL ideology.

## Conclusion and Implication

Identity is an indispensible resource for a teacher agency in the educational context (Vähäsantanen, 2015). However, teacher identity is often replete with tensions, stemming from the challenges of new curriculum, the inconsistency between individual beliefs of teaching and the mandates of the reform, and between the established roles and the roles expected, and potential unfavorable power relations embedded in the reform climate. To reshape and refashion one’s identity in the reform context is a process of ethical self-formation, which requires conducting the work on the self by the self (Clarke, 2009b). Through the lens of the ethico-political diagram of identity work, this study explored how two university English teachers have acted as agents of change, engaging in practices of “work on the self” that have distanced themselves from their previous identities as expert EGP teachers and nurtured their reflective and ethical responsiveness to the challenges of the new ESP curriculum. While navigating the reform, teachers do identity work as ethical self-formation, which involves elements that are relatively stable and those that are evolving. The relatively stable elements function as the catalyst for the other elements to change influenced by the context. Concomitantly, renegotiating identity and exercising an ethical agency to make changes are contingent on teachers’ continuous reflection on their past and future, and critical responsiveness to the new discourses and power dynamics.

The findings of this case study bear several implications for language teachers, university administrators, and policy makers. First, it is imperative for language teachers, teaching either GEP or ESP, to foster reflectivity and the ethical agency in their professional life. Teachers can use Clarke (2009a) framework of the ethico-politics of identity work as a heuristic guide to reflect on their own identities. Specifically, they can regularly take stock of the substance, the authority sources, the practices, and the telos of their identity so as to reflect on the core aspects of their identity, the standards or criterion they follow to make judgment about their work, and their ultimate professional goals. In so doing, it is possible for language teachers to be aware of the limitation of their established identities and practices, come out of their previous methodological and identity comfort zone, open up to alternative possibilities, and be critical to the discursive power relations of the workplace and the application of educational policies. Then, they may engage in “reflective, action-oriented identity practices” as well as becoming “ethical subjects acting on others” in the turbulence of educational change (Miller et al., 2017, p. 91). Moreover, echoing the call from research on similar topics (Jiang et al., 2020; Gao and Cui, 2021), university administrators and policy makers should respect and facilitate the teacher agency through advocating the equality of disciplinary status, attending to teachers’ practical needs, and promoting teacher collaboration. Last but not least, to expect teachers to make changes, there should be system changes too (Vähäsantanen, 2015). One advisable system change could be to establish a balance between requiring teachers’ accountability and allowing teachers’ professional autonomy (Sachs, 2016). In the reform context, this means, while teachers are obliged to adapt their teaching to the new curriculum requirements, they should also be granted room to make adaptation based on their own reflection, agency, and action.

## Data Availability Statement

The raw data supporting the conclusion of this article will be made available by the authors, without undue reservation.

## Ethics Statement

The studies involving human participants were reviewed and approved by Northeast Normal University. The patients/participants provided their written informed consent to participate in this study. Written informed consent was obtained from the individual(s) for the publication of any potentially identifiable images or data included in this article.

## Author Contributions

The author listed has done this work and approved it for publication.

## Conflict of Interest

The author declares that the research was conducted in the absence of any commercial or financial relationships that could be construed as a potential conflict of interest.

## Publisher’s Note

All claims expressed in this article are solely those of the authors and do not necessarily represent those of their affiliated organizations, or those of the publisher, the editors and the reviewers. Any product that may be evaluated in this article, or claim that may be made by its manufacturer, is not guaranteed or endorsed by the publisher.
