# Supplementary material for: Identity Work as Ethical Self-Formation: The Case of Two Chinese English-as-Foreign-Language Teachers in the Context of Curriculum Reform
Source: Front Psychol. 2022 Jan 10;12:774759. doi: 10.3389/fpsyg.2021.774759 (PMC8784658; doi:10.3389/fpsyg.2021.774759)
Supplement: Supplementary file 1 [file Data_Sheet_1.pdf]

APPENDIX

Narrative Frame for EGP Teaching Experiences

1. Reflecting on my EGP teaching experiences, I have constituted myself as a teacher with…
2. When I taught EGP, I used to …
3. At that time I was a teacher who…
4. This is because I believed …
5. I remember when I taught EGP, I …
6. At that time my goal as an EGP teacher was…

Narrative Frame for ESP Teaching Experiences

1. Since I started to teach ESP, I have constituted myself as a teacher with…
2. Now I am a teacher who…
3. This is because I believe…
4. When I teach ESP, I…
5. Now my goal as an ESP teacher is…
6. I believe that in order to be a qualified ESP teacher, I…
7. In the past when teaching EGP, I aimed to…Now my goal is…I hope in the future…

Example Interview Questions:

1. When you taught GE, what and how would you teach? Can you give some examples?
2. In your recent ESP classes, what and how do you teach? Can you give some examples?
3. What are the episodes, events or experiences that impress you most when you taught GE? What are they when you teach ESP?
4. How would you comment on your job as a GE teacher? How would comment on your job as an ESP teacher?
5. When you taught GE, how did you engage in professional learning in order to improve your teaching? How about shifting to ESP, how do you learn to improve yourself when you teach ESP?
6. What do you think is important for GE teaching? What were the standards that you would follow to evaluate your GE teaching practice?
7. What do you think is important for ESP teaching? What are the standards that you will follow to evaluate your ESP teaching practice?
8. What do you think is your role as an ESP teacher? Is there any difference between your role as an ESP teacher and your role as a GE teacher?
9. What was your goal as a GE teacher? Has your goal changed shifting to ESP teaching?
10. How do you imagine your future development or goals as a teacher?

Themes, Codes, and Example Quotes of the Interviews

Theme	Codes	Example Quotes
The substance of teacher identity	Teachers being knowledge authority	I would say I am a qualified teacher, or a knowledge authority. (Wang Interview 1 early Year 1)
	Teaching as a meaningful endeavour	Whereas as a teacher, I hope I can orient students to continue their will willingly, which will make me feel I'm doing something really meaningful. (Zhao Interview 2 end of Year 1)
The authority sources of teacher identity	Transmitting knowledge and training skills (GE)	[When teaching intensive reading], I prioritized teaching language knowledge. As a teacher, I think it necessary to help students set a solid foundation. (Zhao Interview 1 early Year 1)
	Catering to students' needs (ESP)	I believe a defining feature of ESP is that it is a needs-driven subject. Our course objectives and design, teaching materials and methods, and even assessment should be structured according to students' real learning needs (Wang Interview 3 middle of Year 2).
The self-practices of teacher identity	Teaching language knowledge (GE)	I usually focused on teaching language knowledge, including usage of key words and phrases, complex sentence structures and patterns, grammar, textual knowledge and so forth. (Zhao Interview 1 early Year 1)
	Learning with/from students (ESP)	I told my students that I will learn with them, and sometimes even from them, because ESP is a challenge for me too, especially in terms of the medical science knowledge it involves.(Zhao interview 2 end of Year 1)
	Collaborating with subject-matter colleagues (ESP)	When I prepare the lessons, I will sometimes consult subject-matter teachers about my lesson plans. I often ask EMI teachers to provide some of their teaching materials, which I will use in my class as extensive reading materials. Students really like this. (Wang Interview 2 end of Year 1)
	Initiating/engaging in expensive learning (ESP)	Since then most of us have been more enthusiastic in learning subject-matter knowledge and collaborating with students and subject-matter colleagues. We have formed an ethos of learning so as to become better ESP

		teachers.(Wang interview 4 end of Year 2)
The telos of teacher identity	Gaining recognition of university authorities (GE)	To be an expert teacher, you must gain the recognition of the university authorities.(Zhao interview1 early Year 1)
	Realizing self-growth and self-worth (ESP)	Now my goal is to realize self-development while catering to students' learning needs. In the future, I hope I can contribute more to the development of ESP as a discipline and enrich my self-worth as an ESP teacher. (Wang interview 4 end of Year 2)
